# miR-155 Directly Targets MARCH7 and Modulates Viability and Invasive Phenotypes in Breast Cancer Cells

**DOI:** 10.3390/ijms27188092

**Published:** 2026-09-11

**Authors:** Xiaohua Chen, Huayun Qiu, Fangying Chen, Yue Cao, Lei Rao

**Affiliations:** 1 Department of Medical Technology, School of Medicine, Shaoguan University, Shaoguan 512026, China; xiaomaomi@student.usm.my (X.C.);; 2 Institute for Research in Molecular Medicine (INFORMM), Universiti Sains Malaysia, Pulau Pinang 11800, Malaysia; 3 Department of Nursing, School of Medicine, Shaoguan University, Shaoguan 512026, China

**Keywords:** miR-155, MARCH7, PTEN, breast cancer, metabolic cell viability, migration, invasion, PI3K/AKT

## Abstract

miR-155 is an oncogenic microRNA implicated in breast cancer, but its downstream post-transcriptional targets remain incompletely defined. This study tested membrane-associated RING-CH 7 (MARCH7) as a direct miR-155 target and examined associated molecular and cellular phenotypes in MCF-7 and MDA-MB-231 breast cancer cells. Basal miR-155 expression was assessed in MCF-10A, MCF-7, and MDA-MB-231 cells. MCF-7 cells were analyzed by MTT, RT-qPCR, Western blotting, bioinformatic prediction, dual-luciferase reporter assays, and MARCH7 knockdown; MDA-MB-231 cells were additionally evaluated by MTT, RT-qPCR, wound-healing, and Transwell invasion assays. miR-155 was more abundant in the breast cancer cell lines than in MCF-10A cells. In MCF-7 cells, miR-155 overexpression increased MTT-derived metabolic viability, reduced MARCH7 and PTEN expression, increased Cyclin D1, reduced cleaved caspase-3, and was associated with increased PI3K and AKT phosphorylation. In MDA-MB-231 cells, RT-qPCR confirmed successful miR-155 modulation, whereas MTT changes were modest; these small changes may reflect either model/endpoint differences or limited sensitivity of the MTT assay under the tested conditions. Dual-luciferase assays confirmed direct targeting of the MARCH7 3′-UTR by miR-155, while miR-155 inhibition markedly reduced MDA-MB-231 migration and invasion. These findings establish MARCH7 as a direct miR-155 target in the models examined. The PTEN/PI3K/AKT changes are associated downstream findings and are not yet proven to be mediated specifically by MARCH7.

## 1. Introduction

Breast cancer is among the most common malignancies affecting women worldwide and remains a major cause of cancer-related morbidity and mortality [1]. Its development is a complex multistep process involving genetic and epigenetic alterations that collectively reshape cell growth, survival, and metastatic behavior [2]. Identifying aberrantly expressed regulatory molecules and defining their downstream effectors are therefore important for understanding breast cancer biology and for developing potential diagnostic and therapeutic targets.

MicroRNAs (miRNAs) are short non-coding RNAs of approximately 21 nucleotides that regulate gene expression predominantly at the post-transcriptional level and participate in diverse physiological and pathological processes [3]. Among the numerous miRNAs implicated in breast cancer, miR-155 was selected for focused investigation because it is one of the most consistently reported oncogenic and inflammation-associated miRNAs in this disease, has been detected at elevated levels in breast cancer tissues and cell models, and has been linked to tumor progression, metastatic behavior, treatment response, and recurrence-related phenotypes [4,5,6]. This choice was further supported by our recent systematic review, currently available as a preprint, which synthesized 37 breast cancer studies evaluating miR-155 in tissue, blood, and urine and highlighted its potential relevance to diagnosis, disease monitoring, treatment response, recurrence, and metastasis prediction [7]. Importantly, previous work in MCF-7 and other breast cancer models has demonstrated functional effects of miR-155 modulation, but its context-dependent downstream targets remain incompletely characterized [8,9,10,11,12]. These observations provided a strong biological and disease-specific rationale for prioritizing miR-155 rather than performing a broad screening of multiple miRNAs in the present mechanistic study.

Membrane-associated RING-CH 7 (MARCH7), a RING-CH-type E3 ubiquitin ligase, was prioritized as the candidate downstream effector for three reasons. First, MARCH7 has established roles in protein turnover and cancer-associated signaling, including regulation of the MDM2-p53 pathway and promotion of proliferative/invasive phenotypes in other malignancies [13,14,15]. Second, our target-prediction workflow identified a conserved miR-155-responsive sequence within the MARCH7 3′-UTR, making it experimentally testable by mutational dual-luciferase analysis. Third, the potential link between an oncogenic miRNA and an E3 ubiquitin ligase provides a biologically plausible mechanism through which post-transcriptional regulation could influence multiple downstream proteins. PTEN, a major negative regulator of PI3K/AKT signaling, was therefore examined as an associated downstream molecular readout rather than being assumed a priori to be a direct biochemical substrate of MARCH7 [16].

This study examined the phenotypic and molecular effects of miR-155 modulation and tested whether MARCH7 is a direct downstream target of miR-155. MCF-7 cells were selected as the principal mechanistic model because they represent a luminal, hormone-receptor-positive breast cancer context and showed a measurable viability response to miR-155 modulation in our preliminary experiments. MDA-MB-231 cells were included as a biologically distinct triple-negative model with strong migratory and invasive behavior. The study was not designed as an exhaustive comparison across all molecular subtypes; therefore, a HER2-positive model such as SK-BR-3 was not included and is identified as an important direction for future validation. MTT-based metabolic viability measurements, migration and invasion assays, molecular analyses, dual-luciferase reporter assays, and MARCH7 knockdown experiments were integrated to evaluate the miR-155/MARCH7 relationship. Because the present experiments do not directly establish a biochemical interaction between MARCH7 and PTEN, PTEN expression and PI3K/AKT phosphorylation are interpreted as associated downstream changes rather than evidence of a complete MARCH7/PTEN signaling axis.

## 2. Results

### 2.1. Basal miR-155 Expression and Verification of Transfection Efficiency

**Figure 1:**
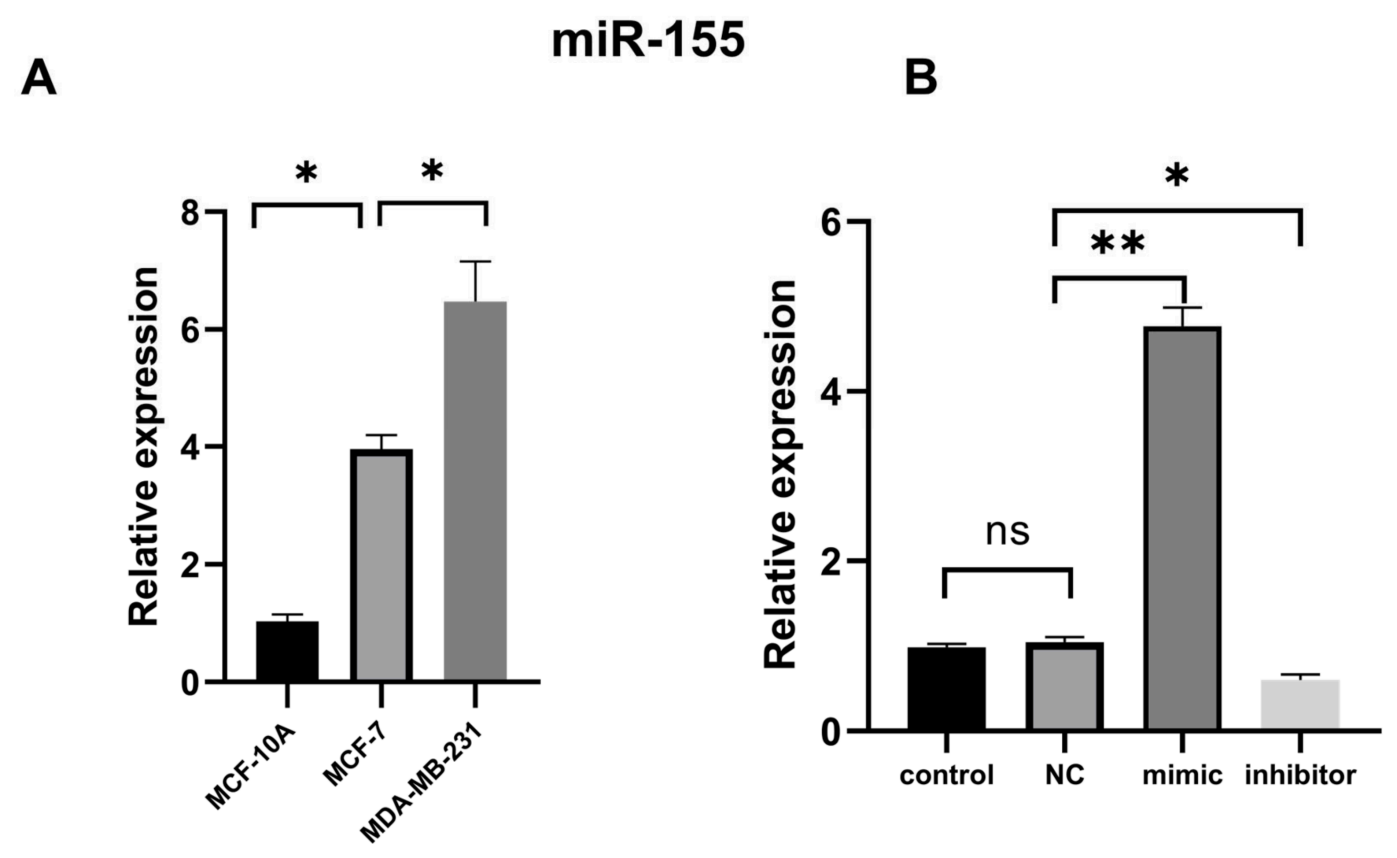
Basal miR-155 expression and verification of transfection efficiency. (**A**) Basal miR-155 expression in MCF-10A, MCF-7, and MDA-MB-231 cells. (**B**) Changes in miR-155 expression in MCF-7 cells 48 h after transfection with 50 nM miR-155 mimic or 100 nM inhibitor and their corresponding negative controls. U6 small nuclear RNA was used as the endogenous control. Data are presented as mean ± SD from three independent experiments. NC, negative control; ns, not significant; * *p* < 0.05; ** *p* < 0.01.

Basal RT-qPCR analysis first showed that miR-155 expression was higher in MCF-7 and MDA-MB-231 breast cancer cells than in non-malignant MCF-10A cells, with the highest basal level observed in MDA-MB-231 cells (Figure 1A). Based on this expression pattern and the distinct biological characteristics of the two cancer cell lines, MCF-7 was used as the principal mechanistic model and MDA-MB-231 as an additional triple-negative model. Transfection efficiency was then confirmed in MCF-7 cells: relative to the corresponding negative controls, 50 nM miR-155 mimic increased miR-155 expression (*p* < 0.01), whereas 100 nM inhibitor decreased its expression (*p* < 0.05) (Figure 1B). U6 small nuclear RNA was used as the endogenous control for miR-155 RT-qPCR.

To explore whether miR-155 modulation was accompanied by changes in other breast cancer-associated miRNAs, we additionally examined miR-16-5p and miR-21-5p by RT-qPCR in MDA-MB-231 cells. The exploratory measurements showed a downward trend in miR-16-5p following miR-155 mimic treatment, whereas miR-21-5p did not show a consistent treatment-associated change. Because this exploratory dataset was limited in sample numbers and showed dispersion among some technical duplicate Ct measurements, these observations are interpreted descriptively rather than as confirmatory evidence of statistical significance. They therefore do not establish direct regulation of miR-16-5p or miR-21-5p by miR-155 or prove that these miRNAs function within the same molecular pathway.

### 2.2. Cell-Line-Dependent Effects of miR-155 Mimic and Inhibitor on Metabolic Cell Viability

**Figure 2:**
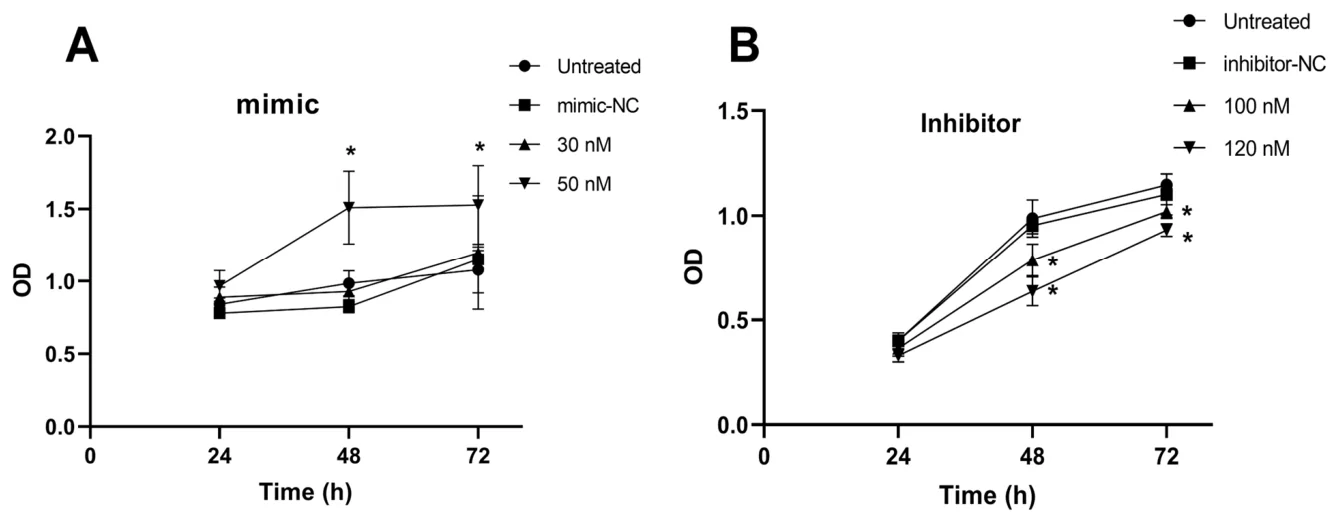
MTT-based metabolic cell viability of MCF-7 cells after miR-155 modulation. (**A**) Cells were treated with 30 or 50 nM miR-155 mimic; the mimic-NC was used at 50 nM. The 50 nM mimic condition showed higher MTT-derived OD values than mimic-NC at 48 h and 72 h. (**B**) Cells were treated with 100 or 120 nM miR-155 inhibitor; the inhibitor-NC was used at 100 nM. Both inhibitor concentrations showed lower OD values than inhibitor-NC at 48 h and 72 h. MTT absorbance is interpreted as an index of metabolic cell viability. Data are mean ± SD from three independent biological replicates. * *p* < 0.05 versus the corresponding negative-control group.

MTT assays were performed over 72 h to assess treatment-associated changes in metabolic cell viability. Differences among groups were limited at 24 h. At 48 h, 50 nM miR-155 mimic produced higher optical-density values than its corresponding 50 nM mimic-NC (*p* < 0.05; Figure 2A), and this difference remained evident at 72 h (*p* < 0.05). These findings indicate an increase in MTT-derived metabolic cell viability following miR-155 mimic treatment under the tested conditions.

In contrast, miR-155 inhibition was associated with lower MTT optical-density values than inhibitor-NC. Both 100 nM and 120 nM inhibitor conditions showed reduced OD values at 48 h and 72 h (*p* < 0.05; Figure 2B). Because 100 nM inhibitor was paired with the concentration-matched 100 nM inhibitor-NC, the 100 nM condition was used as the principal inhibitor comparison for subsequent mechanistic analyses, with 48 h selected as the main molecular-analysis time point.

To determine whether the metabolic response was reproduced in another breast cancer subtype, MDA-MB-231 cells were also examined by MTT after miR-155 modulation. At 48 h, the response was substantially weaker than in MCF-7 cells: the mean optical-density value after 100 nM miR-155 mimic was 1.064 compared with 1.103 in the mimic-NC group (approximately 3.5% lower), whereas 160 nM miR-155 inhibitor produced a mean optical-density value of 1.159 compared with 1.145 in the inhibitor-NC group (approximately 1.2% higher). Thus, neither treatment produced a pronounced separation from its corresponding negative control under these conditions. Together with the RT-qPCR evidence of successful miR-155 modulation, these findings indicate that MDA-MB-231 cells were less responsive than MCF-7 cells to the viability-related effects of miR-155 mimic/inhibitor treatment.

### 2.3. miR-155 Modulation Is Associated with Changes in MARCH7, PTEN, Cyclin D1, and Cleaved Caspase-3

**Figure 3:**
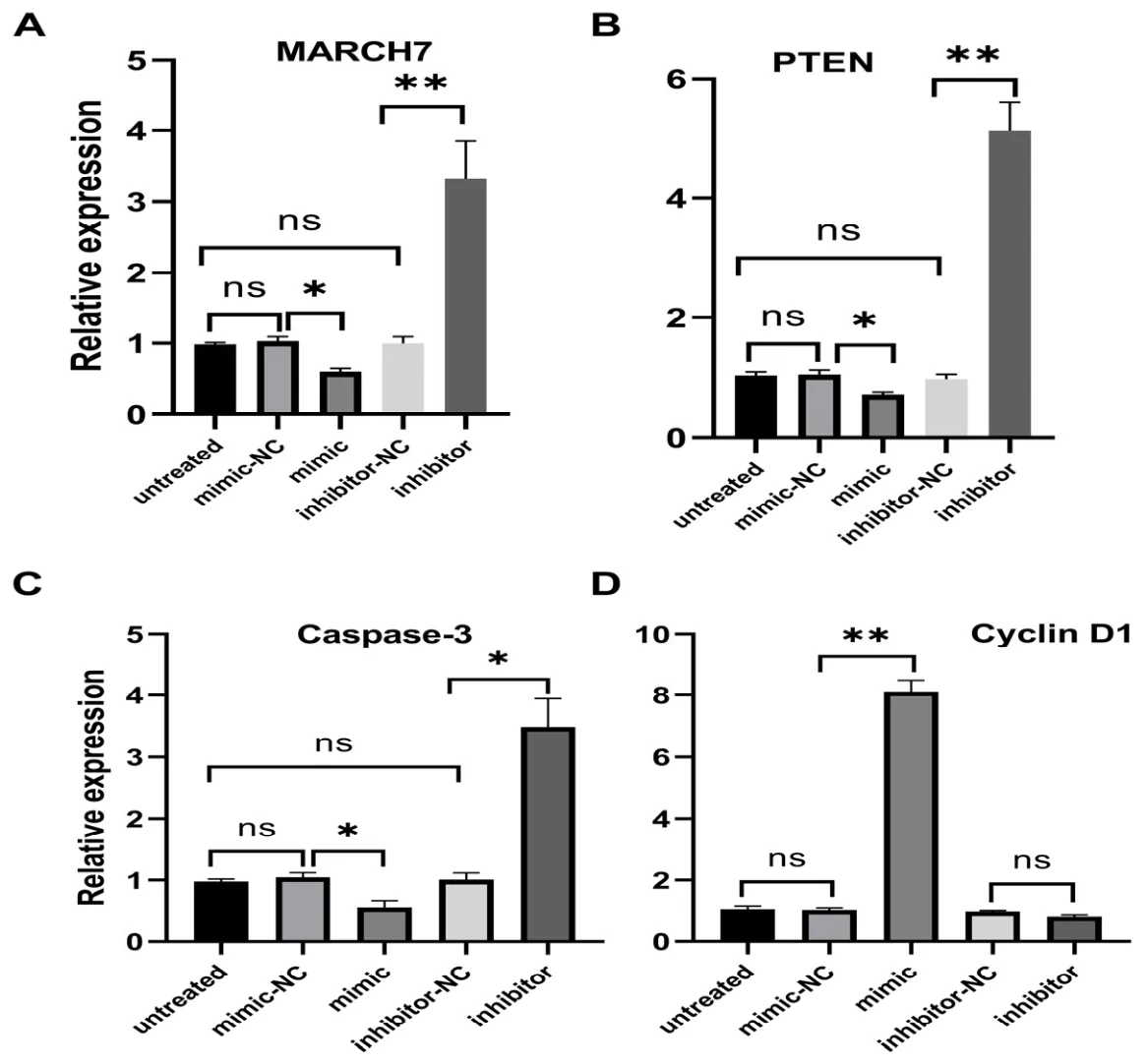
RT-qPCR analysis of (**A**) MARCH7, (**B**) PTEN, (**C**) CASP3, and (**D**) Cyclin D1 mRNA expression in MCF-7 cells 48 h after transfection with 50 nM miR-155 mimic or 100 nM miR-155 inhibitor. The mimic condition decreased MARCH7, PTEN, and CASP3 and increased Cyclin D1 relative to the matched negative control, whereas the inhibitor generally produced the opposite effect for MARCH7, PTEN, and CASP3. GAPDH was used as the endogenous control for mRNA targets [12]. Data are mean ± SD from three independent experiments. ns, not significant; * *p* < 0.05; ** *p* < 0.01.

**Figure 4:**
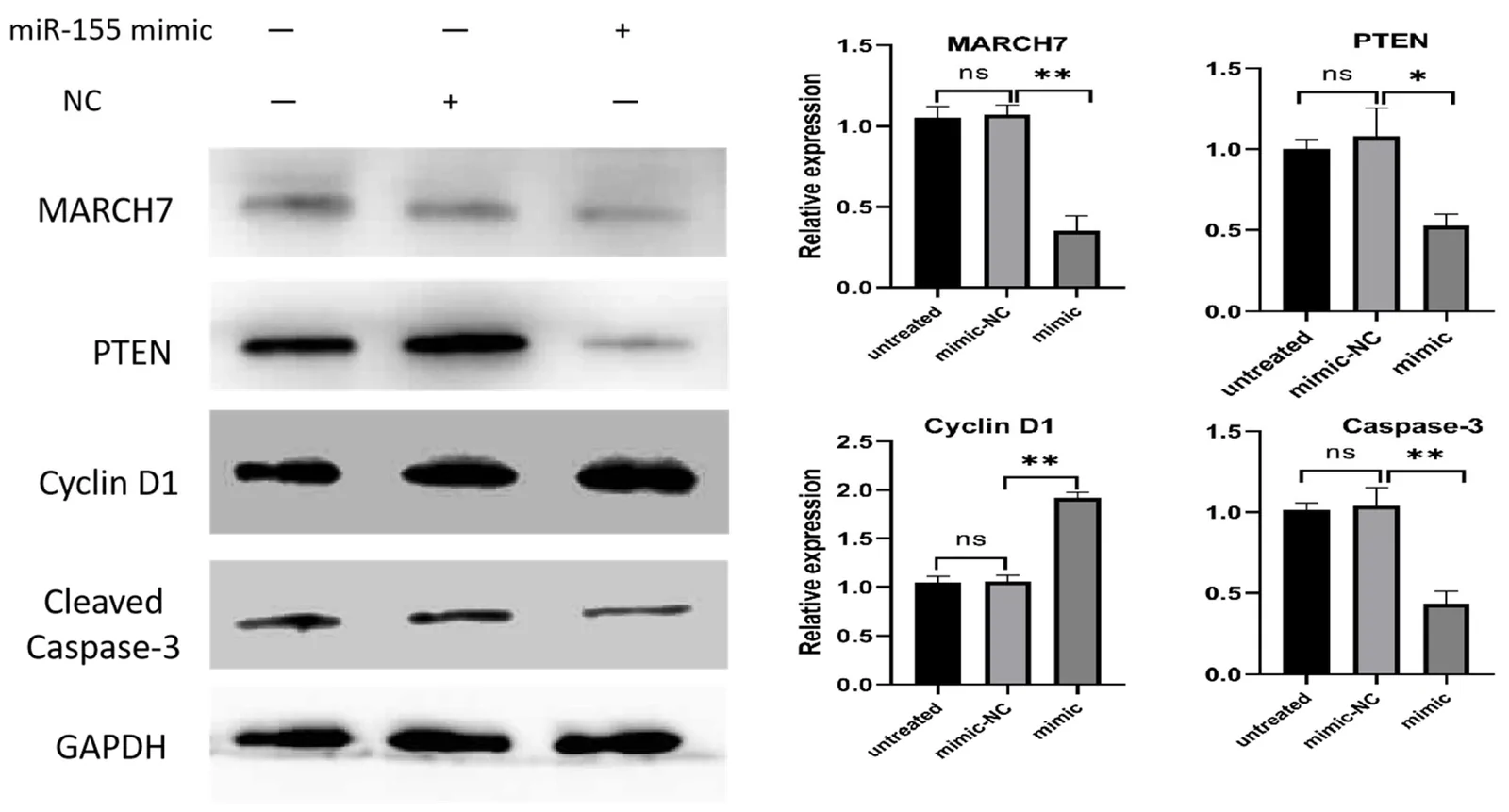
Western blot analysis of MARCH7, PTEN, Cyclin D1, and cleaved caspase-3 in MCF-7 cells 48 h after transfection with 50 nM miR-155 mimic. miR-155 overexpression was associated with lower MARCH7, PTEN, and cleaved caspase-3 abundance and higher Cyclin D1 abundance, consistent in direction with the mimic-associated transcript changes shown in Figure 3. Data are mean ± SD from three independent experiments. ns, not significant; * *p* < 0.05; ** *p* < 0.01.

Bioinformatic prediction identified MARCH7 as a candidate target of miR-155 (Table 1). The RT-qPCR and Western blot datasets showed concordant directions for the mimic condition. Specifically, in MCF-7 cells, miR-155 mimic decreased MARCH7 and PTEN mRNA expression (Figure 3A,B) and likewise decreased MARCH7 and PTEN protein abundance (Figure 4). CASP3 mRNA and cleaved caspase-3 protein were also reduced in the mimic condition, whereas Cyclin D1 increased at both the transcript and protein levels. In contrast, miR-155 inhibition produced the opposite mRNA pattern for MARCH7, PTEN, and CASP3 in Figure 3. Thus, Figure 3 does not show an increase in these markers after miR-155 mimic; the higher bars correspond to the inhibitor condition. These observations support coordinated miR-155-associated molecular changes while not by themselves establishing a complete causal MARCH7/PTEN signaling axis.

### 2.4. MARCH7 Is a Direct Target of miR-155

**Figure 5:**
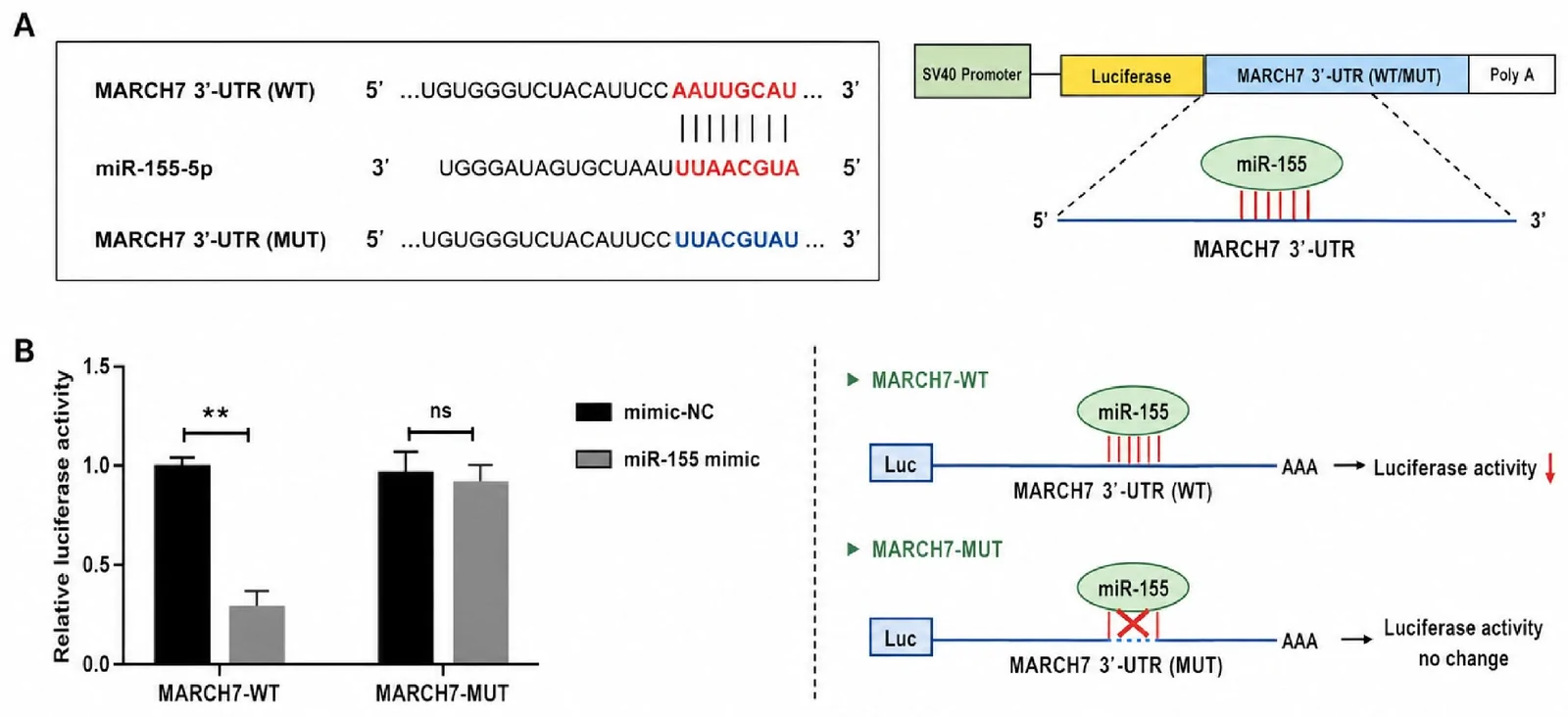
Bioinformatic prediction and dual-luciferase reporter assay confirming MARCH7 as a direct target of miR-155. (**A**) Predicted miR-155 binding site in the MARCH7 3′-UTR and schematic representation of wild-type and mutant reporter constructs. (**B**) Relative luciferase activity after co-transfection with miR-155 mimic or mimic-NC. ns, not significant; ** *p* < 0.01. In panel (**A**), dotted lines indicate predicted sequence complementarity between miR-155-5p and the MARCH7 3′-UTR. Red nucleotides indicate the wild-type seed-matching sequence, whereas blue nucleotides indicate the substituted nucleotides in the mutant construct. The colored boxes represent the labeled components of the reporter vector. The downward red arrow indicates reduced luciferase activity, whereas the red cross indicates disruption of the predicted binding site.

TargetScan alignment predicted a conserved binding site between miR-155 and the MARCH7 3′-UTR (Figure 5A). To validate this direct regulatory relationship, luciferase reporter vectors containing wild-type (WT) or mutant (MUT) MARCH7 3′-UTR sequences were constructed and co-transfected into cells. The dual-luciferase reporter assay demonstrated that the miR-155 mimic significantly suppressed luciferase activity of the MARCH7-WT reporter, whereas this inhibitory effect was completely abolished in the MARCH7-MUT group. Combined with the downregulation of endogenous MARCH7 observed via Western blotting, these findings establish that MARCH7 is a direct downstream target of miR-155, which likely suppresses MARCH7 expression through sequence-specific translational inhibition or mRNA degradation (Figure 5B).

### 2.5. Epistasis Analysis of MARCH7 Knockdown in the miR-155-Inhibited Background

**Figure 6:**
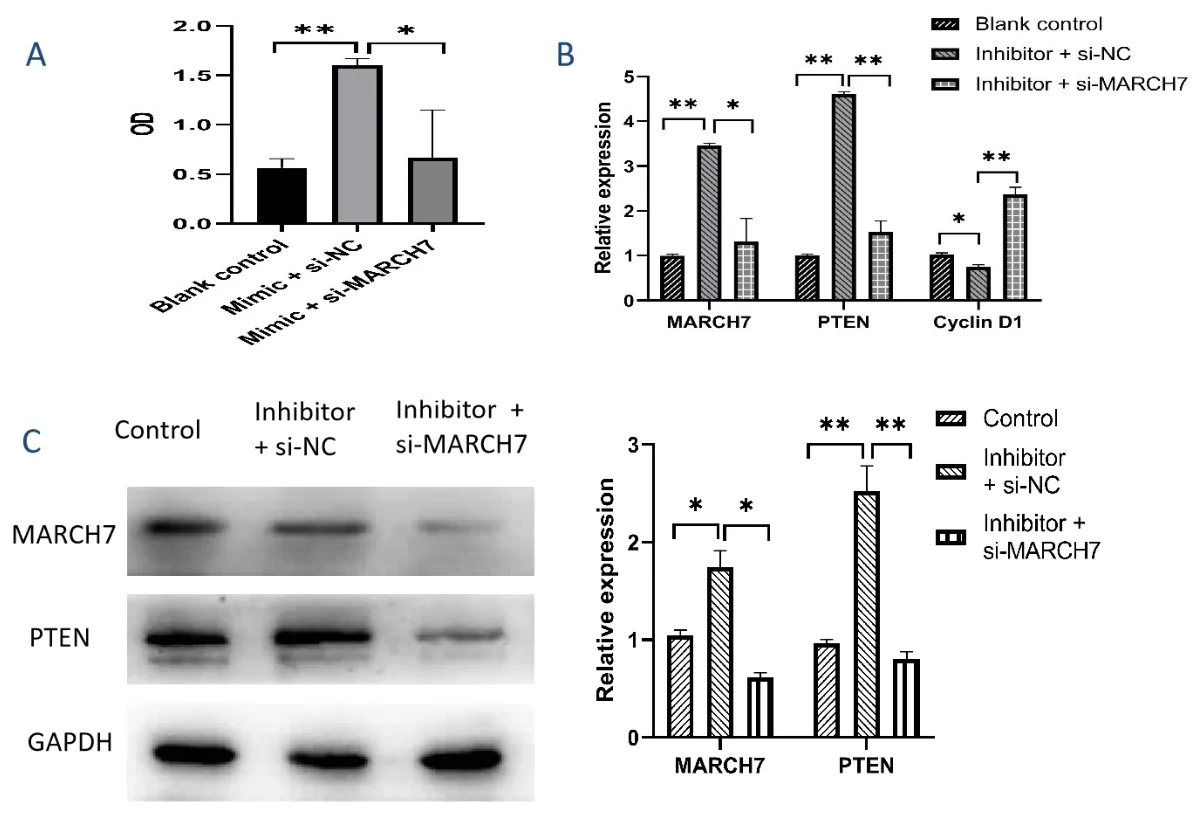
Epistasis analysis of MARCH7 knockdown in MCF-7 cells treated with miR-155 inhibitor. (**A**) MTT-based metabolic cell viability at 48 h. (**B**) RT-qPCR analysis of MARCH7, PTEN, and Cyclin D1. (**C**) Western blot analysis of MARCH7 and PTEN. The inhibitor + si-MARCH7 condition represents a double perturbation and is interpreted as an epistasis experiment rather than a rescue. Because an si-MARCH7-alone arm and a miR-155-insensitive MARCH7 add-back construct were not included, the experiment does not establish a complete causal pathway. Data are mean ± SD from three independent experiments. ns, not significant; * *p* < 0.05; ** *p* < 0.01.

To test whether MARCH7 depletion modifies the phenotype produced by miR-155 inhibition, we performed a double-perturbation (epistasis) experiment in which si-MARCH7 was introduced in the miR-155-inhibited background. Relative to inhibitor + si-NC, inhibitor + si-MARCH7 reduced MARCH7 and PTEN expression, altered Cyclin D1 expression, and was accompanied by partial recovery of MTT-derived metabolic viability (Figure 6). This result indicates that MARCH7 depletion can modify selected readouts in the miR-155-inhibited state. However, because the experiment lacks an si-MARCH7-alone arm and does not use a miR-155-insensitive MARCH7 add-back construct, it cannot isolate the specific contribution of MARCH7 from other miR-155 targets and should not be interpreted as a rescue or definitive mechanistic confirmation.

### 2.6. miR-155 Modulation Is Associated with Changes in PI3K/AKT Phosphorylation

**Figure 7:**
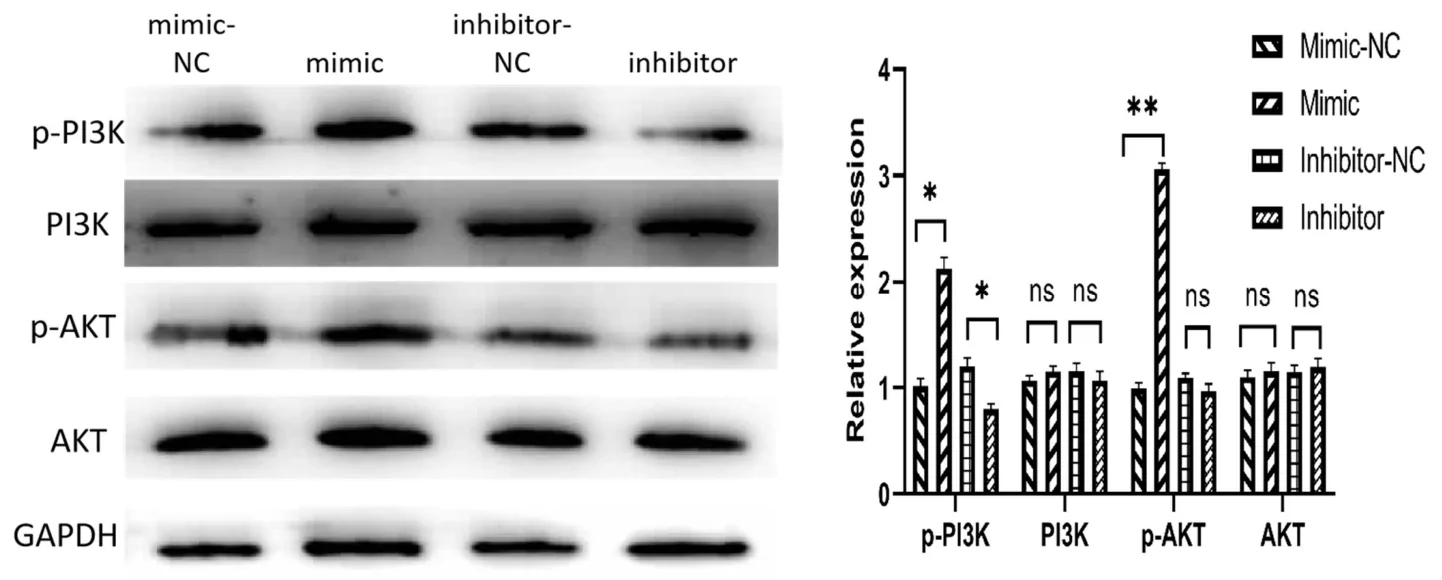
Western blot analysis of PI3K/AKT pathway-related proteins in MCF-7 cells 48 h after treatment with 50 nM miR-155 mimic or 100 nM miR-155 inhibitor. p-PI3K, PI3K, p-AKT, and AKT are shown. Changes in phosphorylated PI3K and AKT are interpreted as changes in signaling activation state, whereas total PI3K and AKT abundance remained comparatively stable. Statistical brackets compare each treatment with its corresponding negative control (mimic versus mimic-NC; inhibitor versus inhibitor-NC). All displayed tested comparisons are explicitly annotated: non-significant comparisons are labeled ns, and significant comparisons are marked with the corresponding asterisks. Data are mean ± SD from three independent experiments. ns, not significant; * *p* < 0.05; ** *p* < 0.01.

Western blotting was used to examine PI3K/AKT pathway proteins in MCF-7 cells. Relative to its matched mimic-NC, 50 nM miR-155 mimic increased p-PI3K and p-AKT, whereas total PI3K and AKT remained largely unchanged (Figure 7). The inhibitor showed the opposite overall phosphorylation trend relative to inhibitor-NC. This pattern is consistent with a change in pathway activation state, because phosphorylation can change rapidly without a corresponding change in total PI3K or AKT abundance over the same experimental interval. Accordingly, the phospho-form changes are interpreted as signaling-state changes associated with miR-155 modulation. They do not demonstrate altered total PI3K/AKT expression and do not establish MARCH7 as the specific mediator of this response.

### 2.7. Inhibitory Effects of miR-155 Suppression on Cell Migration and Invasion

**Figure 8:**
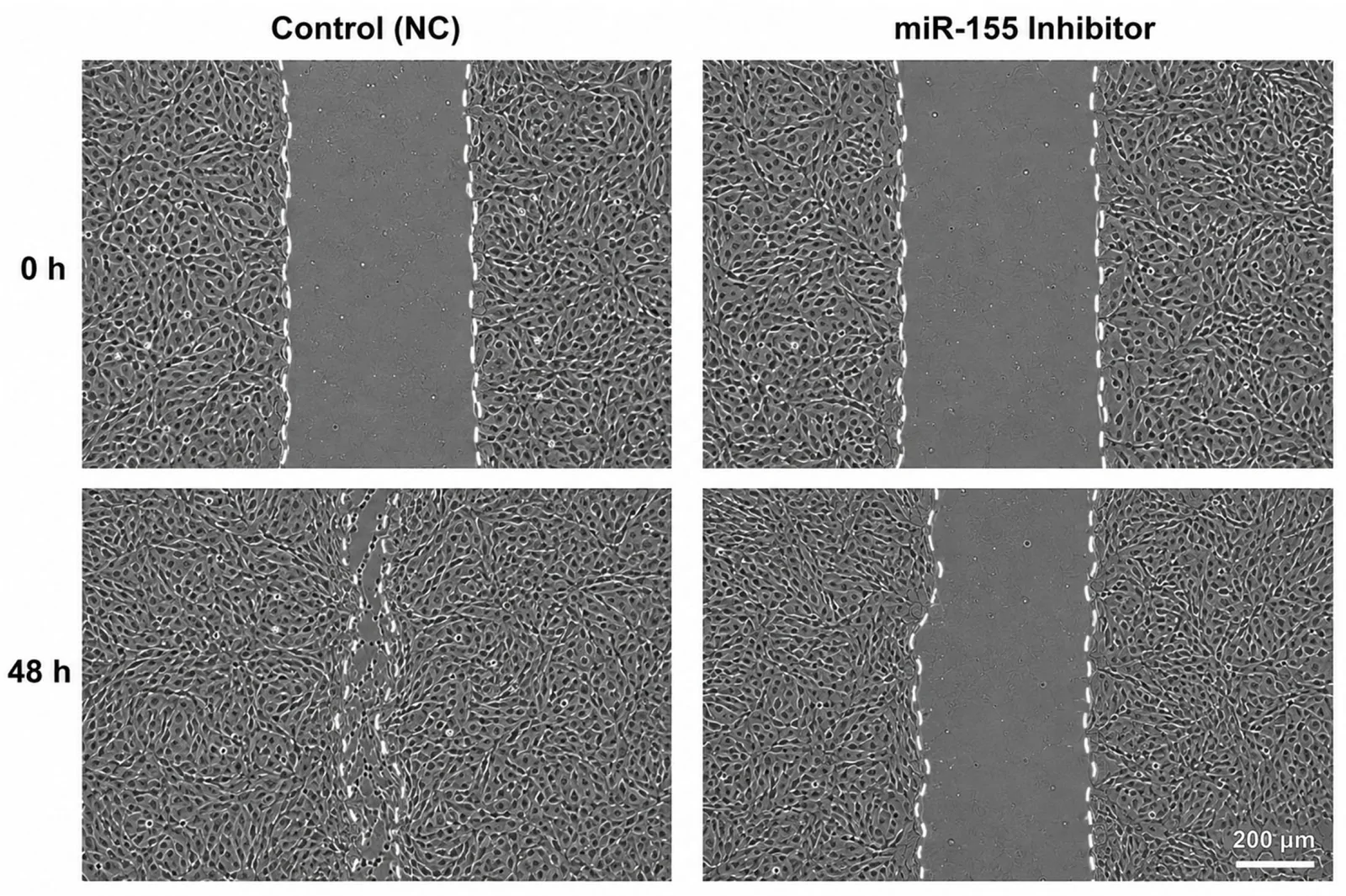
Wound-healing assay in MDA-MB-231 cells after miR-155 inhibition. Representative phase-contrast micrographs are shown at 0 h and 48 h. The representative fields were re-selected from the original microscopy images to provide clearer cellular morphology and wound boundaries. MDA-MB-231 cells were selected for this assay because of their strong migratory phenotype. Dashed lines indicate wound boundaries. Scale bar = 200 micrometers.

**Figure 9:**
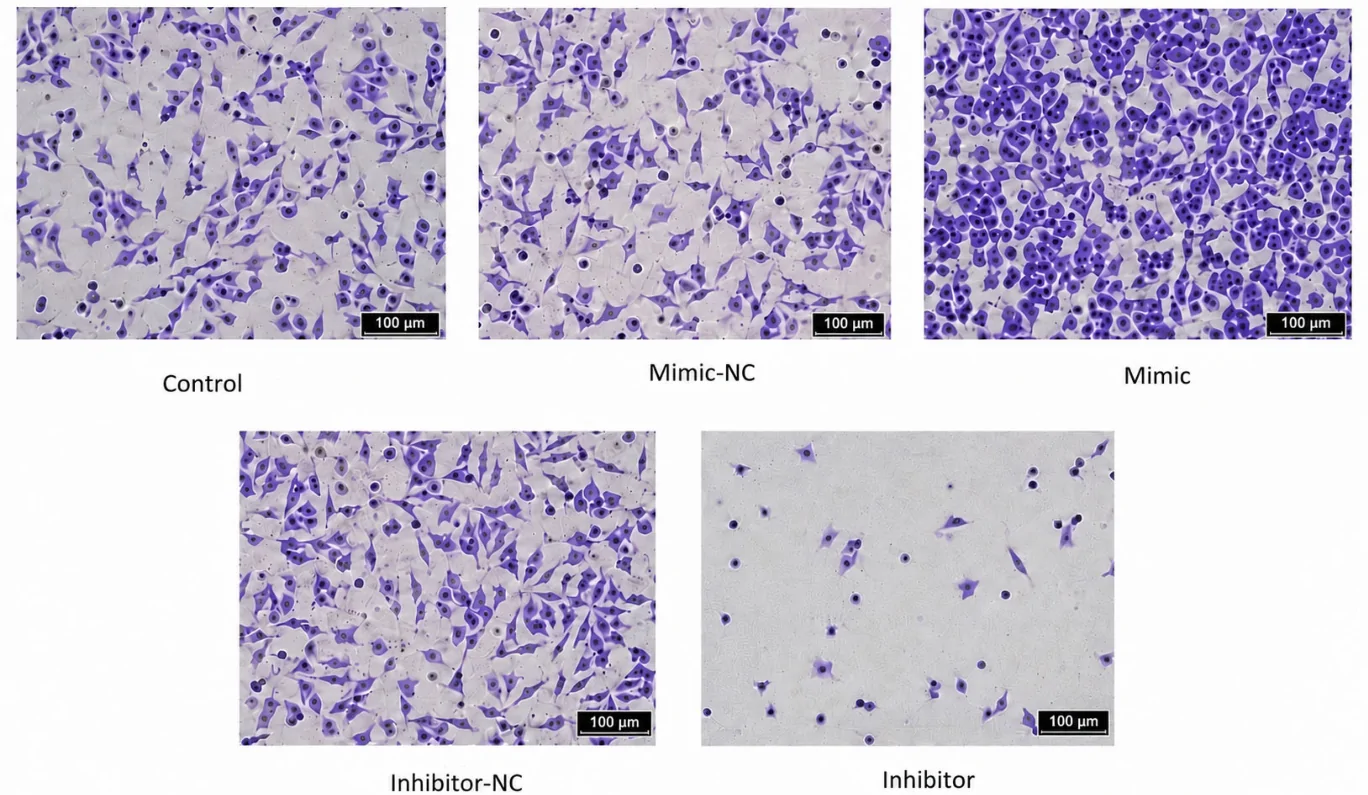
Transwell invasion assay in MDA-MB-231 cells after miR-155 inhibitor treatment. Representative images are shown at ×200 magnification; scale bar = 100 μm.

MDA-MB-231 cells were selected for wound-healing and Transwell assays because this triple-negative line has a substantially more migratory and invasive phenotype than MCF-7 and was therefore used as the motility-focused model. At 48 h, the wound-healing assay showed a lower wound-closure rate after miR-155 inhibitor treatment than in the control group (Figure 8; Table 2), and the Transwell assay showed fewer invading cells in the inhibitor group than in the negative-control group (Figure 9; Table 3). These data are interpreted specifically for MDA-MB-231 cells and are not extrapolated to MCF-7. The different response patterns across MCF-7 and MDA-MB-231 further emphasize cell-line and endpoint heterogeneity in miR-155 biology.

## 3. Discussion

miR-155 has previously been implicated in breast cancer growth, migration, invasion, therapy response, and PI3K/AKT-related signaling [8]. The present study extends this literature by directly validating MARCH7 as a miR-155-responsive 3′-UTR target. The mechanistic protein-level analyses were conducted principally in MCF-7 cells. MDA-MB-231 cells served as a biologically distinct secondary model for miR-155 modulation, metabolic viability, migration, and invasion, but the MARCH7/PTEN/Cyclin D1/cleaved caspase-3 protein panel was not characterized in this line. Moreover, the small MTT changes in MDA-MB-231 cannot by themselves distinguish genuine endpoint-specific biology from limited assay sensitivity. We therefore avoid treating the two cell lines as equivalent mechanistic validation models and restrict cross-line conclusions accordingly.

MARCH7 is an E3 ubiquitin ligase implicated in malignant progression in several cancer types [13,14,15,17]. Our rationale for selecting MARCH7 was based on the convergence of target prediction, its cancer-related ubiquitin-ligase biology, and the availability of a testable miR-155 seed-matching sequence in the MARCH7 3′-UTR. Bioinformatic prediction followed by dual-luciferase reporter analysis showed that mutation of the predicted site abolished miR-155-mediated repression, while endogenous MARCH7 decreased after miR-155 mimic treatment. These complementary observations support MARCH7 as a direct downstream target of miR-155. Public resources also indicate that MARCH7/MARCHF7 is expressed in human breast tissue and that MARCH7 transcripts are represented in primary breast cancer transcriptomic datasets, providing a rationale for future validation in clinical specimens [18,19].

miR-16-5p and miR-21-5p were selected for exploratory analysis because both have established relevance to breast cancer biology: miR-16-5p has been reported to regulate proliferation, invasion, and apoptosis in breast cancer models, whereas miR-21 is a well-characterized oncogenic miRNA associated with breast cancer progression and therapeutic response [20,21]. In the present MDA-MB-231 exploratory assay, miR-16-5p showed a downward trend after miR-155 mimic treatment, whereas miR-21-5p did not show a consistent treatment-associated change. Given the limited sample number and dispersion in some technical duplicate Ct measurements, these findings are hypothesis-generating only. They do not establish direct miR-155-to-miR-16/miR-21 regulation or shared pathway membership. A larger independent biological series, integrated target-network analysis, and direct functional perturbation will be required to determine whether the apparent miR-16-5p trend is reproducible and mechanistically relevant.

PTEN is a major negative regulator of PI3K/AKT signaling [22]. In this study, PTEN expression changed in parallel with MARCH7 following miR-155 modulation, and PI3K/AKT phosphorylation also changed. However, these observations do not establish a direct functional or biochemical MARCH7-PTEN relationship. Figure 6 is an epistasis/double-perturbation experiment showing that MARCH7 depletion within a miR-155-inhibited background modifies PTEN and viability-related readouts, but it does not isolate MARCH7 from other miR-155 targets. Likewise, the phosphorylation data were not obtained after selective MARCH7-alone manipulation. Therefore, the requested MARCH7-alone mechanistic validation remains unresolved in the present study, and the current evidence supports direct miR-155 targeting of MARCH7 together with associated PTEN/PI3K/AKT changes, while the causal link from MARCH7 to PTEN/PI3K/AKT remains open and requires direct validation.

Future work should address three complementary levels of validation. First, the miR-155/MARCH7 relationship should be tested across additional molecular subtypes, particularly a HER2-positive model such as SK-BR-3, and the principal MCF-7 signaling experiments should be replicated in MDA-MB-231 cells while migration assays are evaluated in an appropriate MCF-7 setting. Second, MARCH7 gain-of-function/re-expression and complete control matrices should be used to determine whether MARCH7 is necessary and sufficient for PTEN/PI3K/AKT-associated effects. Third, clinical and in silico studies should examine MARCH7 expression across breast cancer molecular subtypes, its relationship with miR-155, outcome, and organ-specific metastatic tropism, and whether additional miRNAs converge on the same regulatory pathways. These analyses would establish whether the cell-line observations have subtype-specific or clinical relevance. In addition, the exploratory miR-16/miR-21 findings should be extended by integrated in silico co-target/pathway analysis and direct functional validation to determine whether miR-16 participates in the miR-155-associated regulatory network.

Several limitations should be emphasized. First, although the revised Figure 2 no longer shows a decline in the untreated or inhibitor-NC groups at 72 h, late-culture conditions such as increasing confluence or nutrient depletion may still influence MTT measurements at extended time points; therefore, the principal mechanistic interpretation remains centered on the 48 h time point. Second, Figure 6 is a double-perturbation epistasis experiment and lacks an si-MARCH7-alone arm and a miR-155-insensitive MARCH7 re-expression construct, limiting causal inference. Third, selective MARCH7-alone manipulation followed by PTEN and p-AKT measurement was not performed; therefore, MARCH7 cannot yet be identified as the specific mediator of the associated PTEN/PI3K/AKT changes, and this mechanistic gap remains an explicit limitation of the present study. Fourth, the principal protein-level mechanism was characterized in MCF-7 cells; MDA-MB-231 cells were phenotyped but were not subjected to the same MARCH7/PTEN/Cyclin D1/cleaved caspase-3 protein analysis. Fifth, the weak MTT response in MDA-MB-231 may reflect either biology or assay sensitivity. Sixth, HER2-positive cells were not included. Finally, in vivo and clinical-cohort validation and systematic analysis of MARCH7 across molecular subtypes and metastatic sites were beyond the scope of the present study. These limitations narrow the conclusions but do not alter the direct dual-luciferase evidence supporting MARCH7 as a miR-155 target.

## 4. Materials and Methods

### 4.1. Ethics Statement

This study involved established human cell lines only and did not involve human participants, prospectively collected human tissue, identifiable private information, or interventions involving human participants. Therefore, institutional ethics committee approval and informed consent were not applicable to the experiments reported in this manuscript.

### 4.2. Materials

The human breast cancer cell lines MCF-7 and MDA-MB-231 and the non-malignant mammary epithelial cell line MCF-10A were obtained from the Cell Bank of the Chinese Academy of Sciences (Shanghai, China). hsa-miR-155 mimic, mimic negative control (mimic-NC), miR-155 inhibitor, inhibitor negative control (inhibitor-NC), and related oligonucleotides were synthesized by RiboBio (Guangzhou, China, catalog no. R10034.11). Human MARCH7 siRNA (si-MARCH7; catalog no. sc-60235) was purchased from Santa Cruz Biotechnology. DMEM, fetal bovine serum (FBS; catalog no. A5256701), and penicillin-streptomycin (catalog no. 15240-062) were obtained from Gibco (Shanghai, China); complete MCF-10A medium was obtained from Procell Biotechnology (Wuhan, China). MTT (catalog no. 475989) was obtained from Calbio Chem (Shanghai, China). TRIzol reagent was obtained from Thermo Fisher (Shanghai, China, catalog no. 15596026); reverse-transcription reagents (catalog no. FSQ-301) and SYBR Green qPCR reagents (catalog no. QPK-201) were obtained from TOYOBO (Shanghai, China). RIPA lysis buffer (catalog no. P0013C), BCA assay reagent (catalog no. P0012), and ECL chemiluminescence reagent (catalog no. P0018AS) were obtained from Beyotime Biotechnology (Shanghai, China). Antibodies are described in Section 4.8, and primer sequences are listed in Table 4.

### 4.3. Cell Culture and Experimental Grouping

MCF-7, MCF-10A, and MDA-MB-231 cells were authenticated/source-verified by the supplying cell bank. MCF-7 was used as the principal luminal breast cancer mechanistic model, whereas MDA-MB-231 was used as an additional triple-negative model for baseline miR-155 expression, MTT, RT-qPCR, wound-healing, and Transwell experiments. A HER2-positive line was not included because the study was designed as a focused two-subtype mechanistic investigation rather than a comprehensive molecular-subtype comparison.

MCF-7 cells were cultured in DMEM supplemented with 10% FBS and 1% penicillin-streptomycin. MCF-10A cells were cultured according to the supplier’s complete-medium protocol. MDA-MB-231 cells were cultured in high-glucose DMEM supplemented with 10% FBS and 1% penicillin-streptomycin. Cells were maintained at 37 °C in a humidified incubator containing 5% CO_2_ and were passaged or used for experiments at approximately 70–80% confluence.

### 4.4. Cell Transfection

Synthetic miR-155 mimics, miR-155 inhibitors, corresponding negative controls, si-NC, and si-MARCH7 were transfected using Lipofectamine 2000/3000 (Thermo Fisher Scientific Inc., Shanghai, China) according to the manufacturer’s instructions. MCF-7 and MDA-MB-231 cells were routinely cultured in DMEM supplemented with 10% fetal bovine serum and 1% penicillin-streptomycin. During the transfection period, antibiotics were omitted from the complete medium in accordance with the transfection protocol. After 6–24 h, the transfection medium was replaced with routine complete medium containing 10% fetal bovine serum and 1% penicillin-streptomycin, and cells were subsequently cultured until the indicated experimental time points. For MCF-7 cells, 50 nM miR-155 mimic and 100 nM miR-155 inhibitor were selected for the principal mechanistic experiments based on preliminary concentration optimization; mimic-NC was used at 50 nM and inhibitor-NC at 100 nM. For the MDA-MB-231 cross-cell-line sensitivity experiment, 100 nM miR-155 mimic and 160 nM miR-155 inhibitor were evaluated at 48 h.

### 4.5. MTT Assay for Metabolic Cell Viability

Based on the concentration–optimization experiments, 50 nM miR-155 mimic and 100 nM miR-155 inhibitor were selected for subsequent MCF-7 experiments. The corresponding negative controls for these final working conditions were 50 nM mimic-NC and 100 nM inhibitor-NC. The 30 nM mimic and 120 nM inhibitor conditions were part of preliminary concentration screening and did not include separate negative-control arms at those exact concentrations. After transfection, cells from each group were seeded into 96-well plates at a density of 5 × 10^3^ cells per well. For MCF-7 cells, metabolic activity was assessed at 24, 48, and 72 h; MDA-MB-231 cells were additionally evaluated at 48 h for cross-cell-line comparison. At each time point, 10 μL of MTT solution was added to each well, followed by incubation for 4 h at 37 °C. The supernatant was discarded, and 150 μL of dimethyl sulfoxide was added to each well. After gentle shaking for 10 min to fully dissolve the formazan crystals, absorbance at 490 nm was measured using a microplate reader (Thermo Fisher Scientific Inc., Shanghai, China).

### 4.6. Bioinformatic Prediction of miR-155 Targets

TargetScanHuman was used to identify the predicted miR-155-5p binding site within the MARCH7 3′-UTR (TargetScanHuman, version 8.0; https://www.targetscan.org/; accessed 1 October 2024). Candidate target information was additionally reviewed using the miRecords integrated resource for miRNA-target interactions [23]. Because miRecords is a legacy database and its web interface is no longer consistently accessible, it is cited here primarily as the resource used in the original screening workflow. A total of 165 candidate target genes passed the multi-tool filtering criteria. The bioinformatics workflow did not generate a numerical ranking for candidate genes; instead, MARCH7 and other targets were prioritized on the basis of predicted miRNA response elements (MREs), sequence conservation, and their biological relevance to cancer-associated regulatory pathways.

Potential target genes regulated by hsa-miR-155-5p were also examined using the Ensembl Genome Browser (GRCh37/hg19 archive; https://grch37.ensembl.org; accessed 1 October 2024). Genomic position and 3′-UTR sequence context were reviewed, and MARCH7 was selected for direct experimental validation because of its predicted miR-155 seed-matching site and its established function as an E3 ubiquitin ligase in cancer-related pathways. No new multi-miRNA pathway-correlation analysis was performed in the present study; systematic identification of additional miRNAs converging on the same pathway is proposed as a future direction.

### 4.7. RT-qPCR

Total RNA was extracted from MCF-7 and MDA-MB-231 cells using TRIzol reagent and reverse-transcribed into cDNA using the indicated reverse-transcription reagents. Quantitative PCR was performed using SYBR Green chemistry. U6 small nuclear RNA was used as the endogenous control for miR-155 and for the exploratory miR-16-5p/miR-21-5p measurements, whereas GAPDH was used for MARCH7, PTEN, CASP3, and Cyclin D1 mRNA normalization, consistent with its use as an endogenous reference in prior miR-155/MCF-7 RT-qPCR studies [12]. For the exploratory MDA-MB-231 miRNA panel, miR-16-5p and miR-21-5p were measured in two independently labeled samples per condition, with duplicate qPCR wells. Relative expression was calculated using the 2^−ΔΔCt^ method. Because this exploratory panel was limited in sample numbers and showed dispersion in some technical duplicate Ct values, it was interpreted descriptively and was not used for confirmatory inferential statistics.

The miR-16-5p and miR-21-5p primer sequences are provided in Table 4. U6 was used as the endogenous control for these exploratory miRNA measurements. PCR product electrophoresis and melting-curve analysis are provided in Figures S1 and S2, respectively.

### 4.8. Western Blotting

Cells were harvested and lysed with RIPA buffer containing protease inhibitors. Total protein concentration was determined using a BCA protein assay kit. Equal amounts of protein were separated by SDS-PAGE and transferred onto PVDF membranes. After blocking with 5% non-fat milk at room temperature, membranes were incubated overnight at 4 °C with primary antibodies against MARCH7 (1:1000, catalog no. 680404), PTEN (1:2000, catalog no. R381415), Cyclin D1 (1:2000, catalog no. RR380999), PI3K (1:1000, catalog no. R25368), p-PI3K (1:2000, catalog no. R382122), AKT (1:5000, catalog no. R23412), p-AKT (1:2000, catalog no. 341790), and GAPDH (1:15,000, catalog no. R380626). The next day, membranes were incubated with HRP-conjugated secondary antibodies at room temperature. Protein bands were visualized using an ECL chemiluminescence detection system.

### 4.9. Dual-Luciferase Reporter Assay

To verify the targeting relationship between miR-155 and MARCH7, luciferase reporter vectors containing the wild-type (WT) or mutant (MUT) MARCH7 3′-untranslated region (3′-UTR) sequence were constructed. Reporter plasmids were co-transfected with miR-155 mimic or mimic-NC into MCF-7 cells. After 48 h of culture, luciferase activity was measured according to the manufacturer’s instructions. Each experiment was repeated three times, and the mean value was used for analysis.

The wild-type (WT) 3′-untranslated region (3′-UTR) of human MARCH7 containing the predicted binding site for miR-155-5p (5′-…UGUGGGUCUACAUUCCAAUUGCAU…-3′) was PCR-amplified and inserted downstream of the SV40-driven firefly luciferase gene in the reporter vector. A mutant (MUT) reporter construct was generated by substituting the core seed-matching nucleotides from 5′-…AAUUGCAU…-3′ (WT) to 5′-…UUACGUAU…-3′ (MUT) via site-directed mutagenesis (Q5 Site-Directed Mutagenesis Kit, New England Biolabs, Beijing, China). Both WT and MUT recombinant constructs were sequence-verified by Sanger sequencing.

For transfection, cells were seeded in 24-well plates and co-transfected with 200 ng of either MARCH7-WT or MARCH7-MUT reporter plasmid, together with 50 nM of miR-155 mimic or mimic-NC using Lipofectamine 3000 (Thermo Fisher Scientific). At 48 h post-transfection, firefly and Renilla luciferase activities were measured using the Dual-Luciferase Reporter Assay System (Promega, Beijing, China, Cat. #E1910) on a luminometer. Firefly luciferase activity was normalized to Renilla luciferase activity. Co-transfection of miR-155 mimic with MARCH7-WT significantly decreased relative luciferase activity (** *p* < 0.01), whereas co-transfection with MARCH7-MUT exhibited no significant change (ns) compared to the mimic-NC group, confirming that miR-155-5p directly targets MARCH7 via this specific 3′-UTR sequence site.

### 4.10. Wound-Healing Assay for Cell Migration

Cells from each group were seeded in 6-well plates and cultured until reaching 90% confluence. A vertical scratch was created in the cell monolayer using a sterile 200 μL pipette tip. After washing three times with PBS to remove cellular debris, the cells were cultured in serum-free DMEM. Images of the scratch area were captured at 0 h and 48 h using an inverted microscope (Nikon, Tokyo, Japan). The wound-closure rate was calculated as: (initial scratch area − final scratch area)/initial scratch area × 100%.

### 4.11. Transwell Invasion Assay

Cell invasion was assessed using Transwell chambers (8 μm pore size; Corning, Suzhou, China) pre-coated with Matrigel (diluted 1:3 in serum-free DMEM; BD Biosciences, Shanghai, China). Approximately 2 × 10^5^ cells in 200 μL of serum-free medium were added to the upper chamber. The lower chamber was filled with 600 μL of complete medium containing 10% FBS as a chemoattractant. After 24–48 h of incubation, cells remaining on the upper surface were removed with a cotton swab. Invaded cells on the lower surface were fixed with 4% paraformaldehyde, stained with 0.1% crystal violet, and counted in five random fields per well under a light microscope.

### 4.12. Statistical Analysis

Statistical analyses were performed using SPSS version 26.0 and GraphPad Prism version 8.0. All experimental procedures were conducted in at least three independent biological replicates. Quantitative data are expressed as the mean ± standard deviation (SD). Prior to inferential analysis, data normality and homogeneity of variance were assessed using the Shapiro–Wilk test and Levene’s test, respectively. Inter-group comparisons between two groups were evaluated using the independent-samples *t*-test. For multi-group comparisons at a single time point, a one-way analysis of variance (ANOVA) was performed, followed by Tukey’s or LSD post hoc tests where appropriate. For MTT data involving multiple time points and groups, a two-way ANOVA was utilized to evaluate the main effects of time, treatment group, and their interaction. Statistical significance was defined as *p* < 0.05.

## 5. Conclusions

This study provides direct dual-luciferase evidence that MARCH7 is a downstream target of miR-155 in the breast cancer cell models examined. In MCF-7 cells, miR-155 modulation was associated with changes in metabolic cell viability, MARCH7 and PTEN expression, PI3K/AKT phosphorylation, and phenotype-related molecular markers. In MDA-MB-231 cells, miR-155 modulation was confirmed by RT-qPCR, MTT changes were modest, and miR-155 inhibition reduced migration and invasion. Because the MDA-MB-231 protein pathway was not characterized and the MTT assay may have limited sensitivity in this line, these findings are interpreted as secondary phenotypic observations rather than cross-cell-line mechanistic confirmation. The Figure 6 double-perturbation experiment indicates epistatic modification of selected readouts but does not constitute a rescue or establish a causal MARCH7/PTEN/PI3K/AKT axis. Selective MARCH7 gain-/loss-of-function followed by PTEN and p-AKT analysis, together with validation in additional breast cancer models, is required to define the downstream mechanism.

## Figures and Tables

**Table 1:** Partial prediction results for candidate miR-155 target genes.

Accession No.	Gene	Accession No.	Gene
NM_022826	*MARCH7*	NM_020403	*PCDH9*
NM_019027	*RBM47*	NM_020661	*AICDA*
NM_018557	*LRP1B*	NM_173667	*FLJ37543*
NM_018243	*SEPT11*	NM_173355	*UPP2*
NM_014904	*RAB11FIP2*	NM_145174	*DNAJB7*
NM_014765	*TOMM20*	NM_058172	*ANTRX2*
NM_014577	*BRD1*	NM_032228	*FAR1*
NM_014112	*TRPS1*	NM_025133	*FBXO11*

Note: A total of 165 candidate genes were identified across the screening databases. Candidate genes were prioritized based on binding site features, sequence conservation, and functional pathways rather than a numerical ranking system.

**Table 2:** Effect of miR-155 inhibitor on scratch healing ability of MDA-MB-231 cells (*n* = 3).

Time	Control	Inhibitor	*F*	*p*
0 h Closure Rate (%)	0.00 ± 0.00	0.00 ± 0.00	-	-
48 h Closure Rate (%)	78.74 ± 3.95	37.52 ± 3.28 **	251.903	<0.001

Note: ** *p* < 0.001 versus the indicated control group. Wound areas were quantified using ImageJ (version 1.54).

**Table 3:** Comparison of invading cell counts across experimental groups (*n* = 3).

Group	Number of Invading Cells	*F*	*p*
Blank Control	88.40 ± 7.12	164.273	<0.001
mimic-NC	90.20 ± 8.06		
mimic	132.60 ± 10.54 **		
inhibitor-NC	91.80 ± 7.94		
inhibitor	21.40 ± 4.36 **		

Note: ** *p* < 0.001 versus NC group. Cell counting was performed by averaging the numbers from 5 randomized high-power fields across 3 independent replicates.

**Table 4:** Primer sequences used for RT-qPCR.

Primer Name	Sequence (5′ to 3′)
hsa-miR-155-5p Forward	GCGGTTAATGCTAATCGTGATA
hsa-miR-155-5p Reverse	ATCCAGTGCAGGGTCCGAGG
U6 Forward	CTCGCTTCGGCAGCACA
U6 Reverse	AACGCTTCACGAATTTGCGT
PTEN Forward	GACGAACTGGTGTAATGATATG
PTEN Reverse	GTGCCACTGGTCTATAATCC
GAPDH Forward	TCTGACTTCAACAGCGACACC
GAPDH Reverse	GTTGCTGTAGCCAAATTCGTT
CASP3 Forward	AGATGTTTGAGCCTGAGCA
CASP3 Reverse	GTGCGTATGGAGAAATGGGC
MARCH7 Forward	CTTCCCGAAGCAATACGCAG
MARCH7 Reverse	TCGTTGTCCGCCTACCTTCA
Cyclin D1 Forward	AGCTGTGCATCTACACCGAC
Cyclin D1 Reverse	GAAATCGTGCGGGGTCATTG
hsa-miR-16-5p Forward	CGCGTAGCAGCACGTAAATA
hsa-miR-16-5p Reverse	AGTGCAGGGTCCGAGGTATT
hsa-miR-21-5p Forward	GCGCGTAGCTTATCAGACTGA
hsa-miR-21-5p Reverse	AGTGCAGGGTCCGAGGTATT

## Data Availability

The original contributions presented in this study are included in the article/Supplementary Material. Further inquiries can be directed to the corresponding author.

## Supplementary Materials

The following supporting information can be downloaded at: https://www.mdpi.com/article/10.3390/ijms27188092/s1.

## Abbreviations

The following abbreviations are used in this manuscript:

miR-155	microRNA-155
MARCH7	Membrane-associated RING-CH 7
PTEN	Phosphatase and tensin homolog
PI3K	Phosphoinositide 3-kinase
AKT	Protein kinase B
RT-qPCR	Reverse transcription quantitative polymerase chain reaction
UTR	Untranslated region
WT	Wild type
MUT	Mutant
NC	Negative control
SD	Standard deviation
